# Strategies to Increase the Percentages of Vaccination Coverage

**DOI:** 10.3390/vaccines10122103

**Published:** 2022-12-08

**Authors:** Pedro Plans-Rubió

**Affiliations:** 1 Public Health Agency of Catalonia, Department of Health of Catalonia, 08005 Barcelona, Spain; pedro.plans@gencat.cat; 2 Ciber of Epidemiology and Public Health (CIBERESP), 28028 Madrid, Spain

In 2012, the WHO proposed the Global Vaccine Action Plan (GVAP) 2011–2020 to promote essential or routine vaccines among all children of the world [1]. National vaccination programs must administer the essential vaccines to children and the recommended vaccines to adolescents, adults and specific population groups [1,2].

Sustained high percentages of vaccination coverage are necessary to achieve high levels of individual protection and sufficient herd immunity levels to successfully control vaccine-preventable diseases [3,4,5]. For this reason, the WHO proposed to achieve at least 90% vaccination coverage with three doses of diphtheria–tetanus–pertussis (DTP) vaccines by 2015 and at least 90% vaccination with all vaccines in national programs by 2020 [6]. However, the percentages of vaccination coverage registered in the target vaccination population in many developed and undeveloped countries remain below these recommended levels [3,4]. 

For the vaccines not included in the GVAP, the percentages of vaccination coverage in the target population groups (adults, high-risk individuals, specific population groups) were also below the recommended levels in most countries. In European countries and in developed countries worldwide, the objective for influenza vaccination is 75% in individuals aged more that 55−65 years, individuals with a high-risk of influenza complications, healthcare professionals, and pregnant women [7]. Nevertheless, in 2018 (or nearest year), the percentages of influenza vaccination coverage reported among individuals aged 65 and over were less than 50% in the majority of European Union countries [8]. 

The question is therefore: How can the percentages of vaccination coverage be increased? The strategies to increase the percentages of vaccination coverage can be focused on the following aspects: resources available for vaccination programs; vaccine hesitancy and confidence; access to vaccines; organization and management of vaccination programs; immunization information systems; and health policy. The articles published in this Special Issue present interventions and suggest strategies to increase the percentages of vaccination coverage. 

Several articles published in this Special Issue were focused on the access to vaccines. Anastasiou et al. [9] found that having to pay out-of-pocket for vaccination was one of the factors associated with a lower influenza vaccination coverage in European citizens aged 55 or older. Kalucha et al. [10] found that having to pay for the influenza vaccine was also one of the reasons for rejecting influenza vaccination among young health-care workers in Poland. Bianchi et al. [11] found that a catch-up vaccination strategy of vaccination during hospitalization increased the vaccination coverage for the diphtheria tetanus and inactivated polio vaccine (DT-IPV) in individuals aged 65 years or more in France. 

Vaccine hesitancy is defined as a “delay in acceptance or refusal of vaccines despite availability of vaccine services” [12]. The factors that have been associated with vaccine hesitancy include the perceived risk of disease, confidence in the vaccine's efficacy, the vaccine's effectiveness and safety, and the accessibility of vaccination [13]. The strategy to increase vaccination coverage by reducing vaccine hesitancy must be based on the implementation of health education and communication activities to reduce vaccination hesitancy and increase vaccination confidence and acceptance [13]. 

Several articles published in this Special Issue were focused on vaccine hesitancy and confidence. Anastasiou et al. [9] found that influenza vaccination was associated with a high knowledge about the effectiveness and safety of the influenza vaccines. Rodriguez-Blanco et al. [14] found that a good perception of the influenza and Tdap (tetanus, diphtheria, pertussis) vaccines administered during pregnancy was associated with vaccination acceptance among pregnant women in Spain. Qamr et al. [15] found that a campaign to increase vaccine confidence among parents increased the vaccination coverage for the typhoid conjugate vaccine in children in Pakistan. Popa et al. [16] found that the lack of vaccine information and the fear of vaccine side effects were important negative factors against vaccination programs in Romania. Guaraldi et al. [17] found that COVID-19 vaccination among type 2 diabetic patients in Italy was associated with having been vaccinated against influenza and a high education level. Constantino et al. [18] found that a positive attitude towards vaccinations among celiac patients in Italy was associated with the belief in the positive return of vaccine-preventable diseases with declining vaccination coverage.

Several of the articles published in this Special Issue were focused on the health policy measures. Walkowiak et al. [19] found that the implementation of strict health policy measures (COVID-19 passport) led to higher percentages of COVID-19 vaccination coverage in Lithuania compared to the vaccination outcomes in Poland.

More resources should be allocated to national vaccination programs to achieve higher percentages of vaccination coverage [3,4,5]. The WHO [20] has recommended the following investments to improve national vaccination programs: (1) invest in the management of national vaccination programs; (2) invest in advanced immunization information systems able to track each person’s immunization status; (3) invest in strategies to immunize under-vaccinated and unvaccinated persons; and (4) invest in modernizing vaccine supply chains.

The articles published in this Special Issue suggest strategies to increase the percentages of vaccination coverage for routine and non-routine vaccines mainly in developed countries. Future research should be focused on the analysis of specific strategies to increase vaccination rates.

## References

[1] World Health Organization (WHO) (2013). Global Vaccine Action Plan 2011−2020.

[2] European Centre for Disease Prevention and Control (ECDC) (2020). Vaccine Scheduler.

[3] Plans-Rubio P. (2020). Are the Objectives Proposed by the WHO for Routine Measles Vaccination Coverage and Population Measles Immunity Sufficient to Achieve Measles Elimination from Europe?. Vaccines.

[4] Plans-Rubió P. (2021). Vaccination Coverage for Routine Vaccines and Herd Immunity Levels against Measles and Pertussis in the World in 2019. Vaccines.

[5] Plans-Rubió P. (2022). Percentages of Vaccination Coverage Required to Establish Herd Immunity against SARS-CoV-2. Vaccines.

[6] World Health Organization (WHO) (2016). Global Routine Immunization Strategies and Practices (GRISP): A Companion Document to the Global Vaccine Action Plan (GVAP).

[7] European Centre for Disease Prevention and Control (ECDC) (2018). Seasonal Influenza Vaccination and Antiviral Use in EU/EEA Member States—Overview of Vaccine Recommendations for 2017–2018 and Vaccination Coverage Rates for 2015–2016 and 2016–2017 Influenza Seasons.

[8] OECD/European Union (2020). Vaccination against influenza among people aged 65 and over. Health at a Glance: Europe 2020.

[9] Anastasiou O.E., Heger D. (2021). Understanding the Influence of Individual and Systemic Factors on Vaccination Take-Up in European Citizens Aged 55 or Older. Vaccines.

[10] Kałucka S., Grzegorczyk-Karolak I. (2021). Barriers Associated with the Uptake Ratio of Seasonal Flu Vaccine and Ways to Improve Influenza Vaccination Coverage among Young Health Care Workers in Poland. Vaccines.

[11] Blanchi S., Vaux J., Toqué J.M., Hery L., Laforest S., Piccoli G.B., Crochette N. (2020). Impact of a Catch-Up Strategy of DT-IPV Vaccination during Hospitalization on Vaccination Coverage among People Over 65 Years of Age in France: The HOSPIVAC Study (Vaccination during Hospitalization). Vaccines.

[12] MacDonald N.E., SAGE Working Group on Vaccine Hesitancy (2015). Vaccine hesitancy: Definition, scope and determinants. Vaccine.

[13] European Centre for Disease Prevention and Control (ECDC) (2017). Catalogue of Interventions Addressing Vaccine Hesitancy.

[14] Rodríguez-Blanco N., Tuells J., Nolasco A. (2020). Influenza Vaccination Experiences of Pregnant Women as a Predictor of the Intention to Become Vaccinated in Future Pregnancies in Spain. Vaccines.

[15] Qamar F.N., Batool R., Qureshi S., Ali M., Sadaf T., Mehmood J., Iqbal K., Sultan A., Duff N., Yousafzai M.T. (2020). Strategies to Improve Coverage of Typhoid Conjugate Vaccine (TCV) Immunization Campaign in Karachi, Pakistan. Vaccines.

[16] Popa G.L., Muntean A.-A., Muntean M.-M., Popa M.I. (2020). Knowledge and Attitudes on Vaccination in Southern Romanians: A Cross-Sectional Questionnaire. Vaccines.

[17] Guaraldi F., Montalti M., Di Valerio Z., Mannucci E., Nreu B., Monami M., Gori D. (2021). Rate and Predictors of Hesitancy toward SARS-CoV-2 Vaccine among Type 2 Diabetic Patients: Results from an Italian Survey. Vaccines.

[18] Costantino A., Michelon M., Roncoroni L., Doneda L., Lombardo V., Costantino C., Vecchi M., Elli L. (2022). Vaccination Status and Attitudes towards Vaccines in a Cohort of Patients with Celiac Disease. Vaccines.

[19] Walkowiak M.P., Walkowiak J.B., Walkowiak D. (2021). COVID-19 Passport as a Factor Determining the Success of National Vaccination Campaigns: Does It Work? The Case of Lithuania vs. Poland. Vaccines.

[20] Strategic Advisory Group of Experts on Immunization (2019). The Global Vaccine Action Plan 2011–2020: Review and Lessons Learned.

